# Using concept mapping to explore why patients become lost to follow up from an antiretroviral therapy program in the Zomba District of Malawi

**DOI:** 10.1186/1472-6963-13-210

**Published:** 2013-06-11

**Authors:** Beth Rachlis, Farah Ahmad, Monique van Lettow, Adamson S Muula, Medson Semba, Donald C Cole

**Affiliations:** 1 Dalla Lana School of Public Health, University of Toronto, Toronto, Canada; 2 School of Health Policy and Management, York University, Toronto, Canada; 3 Dignitas International, Zomba, Malawi; 4 College of Medicine, University of Malawi, Blantyre, Malawi; 5 District Health Office, Zomba, Malawi

**Keywords:** Concept mapping, HIV/AIDS, Antiretroviral therapy (ART), Losses to follow-up, Malawi

## Abstract

**Background:**

Retention in antiretroviral therapy (ART) programmes remains a challenge in many settings including Malawi, in part due to high numbers of losses to follow-up. Concept Mapping (CM), a mix-method participatory approach, was used to explore why patients on ART are lost to follow-up (LTFU) by identifying: 1) factors that influence patient losses to follow-up and 2) barriers to effective and efficient tracing in Zomba, Malawi.

**Methods:**

CM sessions (brainstorming, sorting and rating, interpretation) were conducted in urban and rural settings in Zomba, Malawi. Participants included ART patients, ART providers, Health Surveillance Assistants, and health managers from the Zomba District Health Office. In brainstorming, participants generated statements in response to “A specific reason why an individual on ART becomes lost to follow-up is…” Participants then sorted and rated the consolidated list of brainstormed items. Analysis included inductive qualitative methods for grouping of data and quantitative cluster identification to produce visual maps which were then interpreted by participants.

**Results:**

In total, 90 individuals brainstormed 371 statements, 64 consolidated statements were sorted (participant n = 46), and rated on importance and feasibility (participant n = 69). A nine-cluster concept map was generated and included both patient- and healthcare-related clusters such as: *Stigma and Fears, Beliefs, Acceptance and Knowledge of ART, Access to ART, Poor Documentation, Social and Financial Support Issues, Health Worker Attitudes, Resources Needed for Effective Tracing,* and *Health Worker Issues Related to Tracing*. Strategies to respond to the clusters were generated in Interpretation.

**Conclusions:**

Multiple patient- and healthcare focused factors influence why patients become LTFU. Findings have implications particularly for programs with limited resources struggling with the retention of ART patients.

## Background

Globally, 34 million people were living with HIV/AIDS by December 2010 and the majority resided in sub-Saharan Africa [1]. In Malawi, the prevalence of HIV is estimated as 11% [2,3] and over 650,000 children have been orphaned by AIDS [4]. By June 2010, approximately 58% of those in need were receiving antiretroviral therapy (ART) [5] although disparities continue to exist in access to treatment options, particularly for individuals living in rural areas [6].

Losses to follow-up (LTFU) from antiretroviral therapy is a major cause of patient attrition [7]. LTFU can be considered a catch-all category for patients who miss scheduled appointments or medication pick-ups (over a period of time) although operational definitions vary [8]. In Malawi, the term ‘LTFU from ART’ refers to a patient who is overdue for their appointment and is not known to have stopped ART, died, or transferred to another facility [9]. Importantly, patients who discontinue treatment are vulnerable to drug resistance, AIDS-related illnesses, and death [7,8,10-12].

While various reasons are known, demonstrating associations between various variables and risk of becoming LTFU remains challenging. Reported reasons for LTFU in Malawi and elsewhere have included: food insecurity [13-15], financial constraints [14,16,17], religious and family influences [14,16,18,19], wanting to access traditional medicine [20], stigma and fear of disclosure of HIV status [10,19-22], living far from clinics and transport-related costs [10,14,18,20,23], and poor patient-provider relationships [10,14,20]. Treatment literacy (e.g., understanding the natural course of treatment and the need for adherence to ART) [24,25], experiencing an improvement in health and believing that treatment is no longer necessary [10] may also matter. Some of these are consistent with the findings of our 2011 systematic review exploring livelihood factors and ART adherence [25] although we were limited by the available literature and could not identify new factors that may be relevant or specific to Malawi. Furthermore, our understanding of how these various factors potentially interact to influence whether a patient becomes LTFU over time, remains rudimentary [21].

Zomba is one of Malawi’s most populated districts (population: 670,500) and is predominantly rural [26]. Within Zomba, HIV prevalence is 14.5% although estimates have varied by location and population group, ranging from 12% in an urban centre to >30% at a rural hospital [27,28]. In collaboration with the Malawi Ministry of Health, Dignitas International (DI), a humanitarian non-governmental organization, has delivered comprehensive HIV/AIDS care in Zomba since 2004, enrolling over 20,000 patients on ART. The decentralization of services to rural areas has led to a rapid increase in the number of people accessing ART in the district.

In order to improve patient retention in DI’s ART program, we sought to comprehensively identify factors that influence why patients become LTFU as well as factors that impede successful tracing efforts.

## Methods

Concept Mapping (CM) is a participatory mixed-methods approach that enables diverse groups of stakeholders to share their ideas, representing them in various quantitatively derived visual concept maps [29,30]. CM has three phases: brainstorming of statements in response to a focus prompt; sorting and rating of the consolidated list of brainstormed statements; and interpretation where participants discuss and interpret the maps identifying priority areas for action. The use of CM in this context was advantageous as our aim was to identify the most important and feasible interventions/strategies that address patient retention [31].

### Sample

To ensure a variety of viewpoints, purposive sampling was used to include a heterogeneous participant population with respect to patient losses to follow-up. Participants were selected based on their previous experience with ART and included patient and provider groups. Patient participants included patients who had become LTFU while on ART as well as expert patients (patients on ART trained to assist with clinical tasks and counselling). Provider participants consisted of ART providers (clinical officers, medical assistants, and nurses), Health Surveillance Assistants (HSAs) involved with patient tracing, and members of the Zomba District Health Office (DHO) health management team involved in ART provision. We sought a balanced mix of participants working or receiving care either at Zomba Central Hospital (centralized) or in one of three decentralized rural clinics that varied in terms of distance to Zomba town and patient volume (small versus large). Separate sessions were held for patient and provider groups to avoid responder bias (e.g., patients may not speak freely or may provide responses they think providers want to hear). The brainstorming and rating sessions were arranged with a larger number of diverse participants to capture heterogeneity whereas sorting and rating sessions were conducted with smaller and more alike participants for homogeneity [32]. Sessions were conducted in English (provider groups) or Chichewa (patient groups). Inclusion criteria included being at least 18 years of age and having experience with ART as a patient, provider, manager, or through tracing. An additional criterion of having at least a secondary school education was applied for sorting which required reading and comprehension skills. All CM activities took place in May-July 2011. All CM activities were facilitated by an experienced Malawian research assistant who was briefly trained in CM methodology and was fluent in both English and Chichewa.

### Recruitment

In collaboration with local partners at DI, the University of Malawi, and the DHO, the research team and a bilingual research assistant distributed a study information sheet (SIS) to potential participants. To reach provider participants, the SIS was distributed to staff at participating clinics and the DHO. To reach the patient population, collaborating providers disseminated the study information to their patients. Interested individuals contacted the research team for further details. Patients who were currently LTFU (i.e. had not been seen in care) were identified through a master list of currently-missing patients, generated by DI (and shared with the DHO). Those patients who were identified as LFTU were then contacted (via phone or in person during tracing with HSAs) and if successfully found, were invited to participate in the study. Willing participants provided informed written consent. Participants recruited in brainstorming were invited to participate in sorting (if met additional criterion), rating, and interpretation. To allow flexibility to participate, both group sessions and one-on-one sessions were conducted for all phases although generally brainstorming and interpretation was done in group sessions to generate discussion. Sessions were conducted in private rooms at participating clinics and at the DHO. At the time of recruitment, all patients were informed that by participating in group sessions, they were disclosing their HIV positive status to the other members attending the session. Furthermore, participants were informed that any concerns or issues they had before, during (any phase), or after the study would be addressed confidentially by the study team as outlined in the SIS and consent form. Snacks were provided and all patient participants received a 1 kg bag of sugar or a chitenje (local fabric) and 200 Malawian Kwacha (approximately $1.20 Canadian at the time of the study) to help cover travel costs. Ethical approval was obtained from the University of Toronto HIV Research Ethics Board and the National Health Sciences Research Committee in Malawi.

### Brainstorming

Our focus prompt was developed in consultation with DI and pilot-tested with patients at Zomba Central Hospital. Participants were prompted to generate statements in response to “*Why do individuals on ART become lost to follow-up over time*?” To help participants understand ‘lost to follow-up’, several descriptions were provided including when patients: stop taking their ART, stop coming for appointments, or can’t be found by health providers. Each statement generated in brainstorming was captured by the facilitator. Overall, 90 participants (ten group sessions; five individual sessions) generated 371 statements.

Once all sessions were completed, statement consolidation was initiated. Statements in Chichewa were first translated into English. Each statement generated was read aloud in sequence with the focus prompt (i.e., A specific reason why an individual on ART becomes lost to follow-up is… *statement generated in brainstorming*). Statements that did not answer the prompt were considered irrelevant and were removed. The remaining statements were organized into broad themes (e.g., tracing issues), duplicates eliminated, and similar statements merged to create a final consolidated list. The refined list of 64 statements was then back-translated into Chichewa for sorting and rating. Translations were verified by a bilingual DI staff member.

### Sorting and rating

Sorting activity was completed individually by 46 participants (six group sessions; eight individual sessions). Each participant received a deck of 64 cards with one statement per card. Participants were instructed to make piles of similar statements in ways that *made sense to them* and to give labels to each pile they created to reflect the statements within. Prior to each session, the facilitator provided an example based on food (e.g. crunchy, soft, sweet, and sour) to explain how different people may sort the same item in different ways. Rating was completed by 69 participants (six group sessions; twenty-three individual sessions). Participants rated each statement using a Likert 5-point scale, along two dimensions:

Importance: (i.e., how important the statement is with respect to why patients become lost to follow-up) (1- not at all important to 5- extremely important, compared to all other statements);

Feasibility: (i.e., how feasible it would be to address this statement in order to prevent patients on ART from becoming lost to follow-up) (1- not at all feasible to 5- extremely feasible, compared to all other statements).

### Analysis and generation of maps

Upon completion of the sorting and rating activities, data were exported into Concept Systems Software (Concept System Program, Concept Mapping Incorporated, 2005) to create a final cluster solution prior to the interpretation session. Using the statistical technique of a similarity index, the program first determined the number of participants who sorted each pair of statements together. Each statement was then located in relation to all others on a two-dimensional point map (statements sorted together frequently were located close together). Next, a cluster concept map was generated from the point map to show how the statements could be organized into clusters with common themes using hierarchical cluster analysis [29]. In this step, the researchers examined several cluster maps by forcing an upper and lower limit to the number of clusters in a map. The team began with the upper limit of 15 clusters and merged one cluster at a time until the lower limit of 5 clusters was reached. They examined changes in the cluster solution at each merging step. The final cluster map was identified through consensus among researchers on the number of clusters that retained the most meaningful detail between clusters. Clusters that are smaller in size indicate that statements that make up the cluster were more similarly grouped by participants during sorting relative to larger sized clusters. A stress value statistic reflecting a goodness-of-fit of the point map to the similarity matrix was calculated. Generally, indices range from 0.15 to 0.35 [29,33]. In the present study, the stress value was 0.28 indicating an acceptable value. Bridge maps were generated to explore the relative agreement on rating variables between clusters and across provider and patient groups.

### Interpretation of maps

Overall, 35 participants interpreted the maps (five group sessions; 3 individual sessions) documenting key findings with the facilitator. Participants discussed the content of the main clusters and reached agreement through consensus on the most appropriate cluster labels out of the several labels generated individually by participants during the sorting activity. Through the examination and comparison of the “Importance” and “Rating” variables for each cluster, the most actionable clusters were identified (e.g. where efforts to address LTFU should be directed). Clusters with a rating value of ≥3 are discussed in detail.

## Results

### Participants

**Figure 1:**
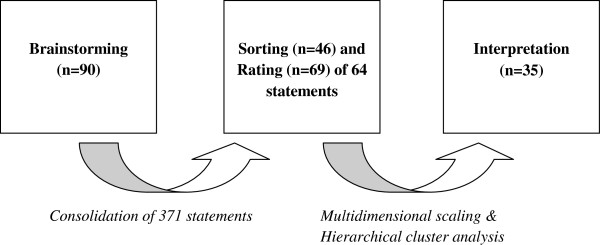
Overview of the concept mapping process.

Participant characteristics are presented in Table 1. Fifty-four percent were women and 49% were under the age of 35 (median: 32, IQR: 27–38). An overview of the CM methods and the number of participants in each stage is presented in Figure 1.

**Table 1:** Number of participants in brainstorming, sorting, rating, and interpretation activities

**Groups**		**# Participants**		
**Task**	**Participant type**	**Urban area**	**Rural area (Pirimiti, Mayaka, Chingale)**	**Total**
**Brainstorming**	Patients	18	23 (including n=9 LTFU)	41
	Providers	29	20	49
**Sorting**	Patients	11	9 (including n = 1 LTFU)	20
	Providers	14	12	26
**Rating**	Patients	14	20 (including n = 2 LTFU)	34
	Providers	17	18	35
**Interpretation**	Patients		20 patients (including n = 1 LTFU)	20
	Providers		15	15

Note: Provider participants included ART providers, Zomba District Health Office Management Team, and Health Surveillance Assistants; LTFU = patient lost to follow-up.

### Cluster solution

**Figure 2:**
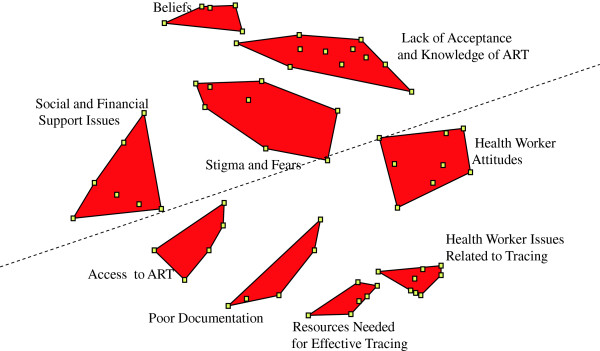
Final nine cluster concept map.

A nine cluster concept map was generated (Figure 2). Clusters included: *Stigma and Fears (8 statements); Beliefs (5 statements); Lack of Knowledge and Acceptance (11 statements); Access to ART (5 statements); Poor Documentation (5 statements); Social and Financial Support Issues (8 statements); Health Worker Attitudes (8 statements); Resources Needed for Effective Tracing (6 statements);* and *Health Worker Issues Related to Tracing (8 statements)*. The map was further divided into two cognitive regions: upper region clusters generally focused on patient-related factors whereas lower region clusters were more healthcare-focused.

### Rating and bridge map

Table 2 presents the mean cluster ratings with associated standard deviations. Table 3 presents all statements. For the rating of “importance” of items, the clusters with the highest rating values were *Poor Documentation, Resources Needed for Effective Tracing,* and *Social and Financial Support Issues*. For the rating of “feasibility” of items to address, the clusters with the highest rating values were *Resources Needed for Effective Tracing, Poor Documentation,* and *Health Worker Issues Related to Tracing*. Generally, ratings were higher for “feasibility” although agreement between the rating variables at the cluster level was relatively high. Patient and providers both gave higher rating values to clusters in the healthcare-related region versus the patient-related region with the exception of *Social and Financial Support Issues.* Provider groups rated clusters in the healthcare-related region as more feasible to address although this pattern was less clear for patient groups (data not shown).

**Table 2:** Cluster characteristics and ratings

**Cluster**	**Importance rating**	**Feasibility rating**
	**Mean (SD)**	**Mean (SD)**
Poor documentation	3.3 (0.18)	3.5 (0.31)
Resources needed for effective tracing	3.3 (0.23)	3.6 (0.14)
Social and financial support issues	3.3 (0.35)	3.4 (0.34)
Health worker attitudes	3.1 (0.25)	3.5 (0.27)
Health worker issues related to tracing	3.1 (0.40)	3.5 (0.20)
Stigma and fears	2.9 (0.44)	3.4 (0.29)
Access to ART	2.7 (0.23)	3.1 (0.22)
Beliefs	2.6 (0.35)	3.0 (0.17)
Lack of knowledge and acceptance	2.6 (0.52)	2.9 (0.33)

**Table 3:** The consolidated list of 64 statements

**Cluster**	**Statements**
Poor documentation	Poor filing means that patient files can get lost when patients on ART are being transferred
	The patient has died but their death is not reported
	Patients have little social support in the villages due to lack of counsellors, support groups and community-based organizations
	The patient has moved away to another clinic without being properly transferred
	Patient visits are not being recorded accurately
Resources needed for effective tracing	Tracing starts too late because of the way a ‘defaulter’ is defined (e.g., missing an appointment by 2 months or more)
	The HSAs have difficulty locating patients because they do not live in their catchment areas and therefore do not know the villages where people come from
	Patients on ART can’t be found if the HSAs do not have their proper address and/or their locator forms
	They live in areas that are difficult to reach so that the HSAs have difficulty tracing them
	Health workers have difficulty locating patients without working phones or phone numbers
	There is no fuel or dedicated transport for tracing
Social and financial support issues	Being too sick to come to the clinic
	The distance to the clinic is too far from some patients on ART
	The guardians of the patient refuse to go the hospital to collect their ARVs from them
	Patients on ART can’t afford transport means to come to the clinic because they face poverty
	A lack of support for ART patients especially when they are orphans
	Patients on ARV medicines may also have to deal with other diseases such as chronic illness
	Patients on ARV medicines feel hungry but they can’t afford to buy extra food
	They are attending to another sick relative (e.g., child)
Health worker attitudes	Patients are on ART feel that there is a lack of confidentiality on behalf of the health workers (e.g., drugs given without privacy)
	Patients on ART get disappointed when they are not put on a different ART regimen to help manage their side effects
	Patients are not properly educated on ART because of little one-on-one counselling with the health workers
	Patients on ART do not like the way they are treated by health workers
	Patients feel shy to come to the health centre because there is limited space and no privacy
	Patients on ART get frustrated because it takes too long before they are seen at the hospital
	Anxiety about going back to the clinic after missing many appointments
	To hide from follow-up, patients change their names and identities
Health worker issues related to tracing	There are too many patients needing to be traced and not enough health workers to trace them
	Poor communication and coordination between the HSA and ART providers
	There is no coordinator for ART tracing and no specific follow-up health workers
	There is no training on how to do the follow-up of ART patients
	The health workers just wait for the patients to come back on their own because their are no consequences for them if they don’t trace them
	There is no deliberate effort to trace ART patients because there are no incentives for the HSAs
	The HSAs do not actually trace patients and instead write fake information about patient visits
	The HSAs don’t value tracing because they have too much other work
Stigma and fears	The patient on ART fears stigmatization because they are transferred to a health centre that is near to where they live
	Fear of divorce if their spouse or loved one discover that they are on ART
	The patient doesn’t want to be associated with ARV drugs because of stigma and fear of isolation
	Patients on ART experience side effects from the medicine
	Patients on ART fear side effects and the unknown
	Patients on ART face mental health issues
	Patients stop coming to the clinic because they get their ARV drugs from somewhere else
	Fear dismissal at the work place because their employer may discover that they are on ART
Access to ART	Patients have difficulty finding ARVs when they travel both within and outside the country
	ARV medicines are not always accessible because of inconsistent drug availability
	Not being able to meet strict hospital policies (e.g., coming with a guardian)
	The patient just picked up a guardian at the market and the guardian cannot be used to trace the patient
	Patients are mobile and they move around a lot
Beliefs	Religion and the belief that prayer, not ARVs, will heal them
	Church fellowships discourage them from taking their ARVs and tell them they are healed from HIV
	Patients on ART believe that they are HIV negative because they gave birth to a negative baby or re-tested negative for HIV
	Beliefs in traditional medicines
	Patients on ART believe that they are healed from HIV if they sleep with a virgin or a younger person
Lack of knowledge and acceptance	Patients feel tired of taking their drugs
	Patients on ART don’t see the need to be on drugs anymore when they feel better and their health has improved
	When there is no improvement in their health, patients on ART are frustrated because they were expecting a quick recovery
	The patient on ART has not accepted their HIV status
	Patients on ART want to have a normal life and feel the medicine is a burden
	Patients on ART are just not serious about their lives
	Patients see no value in taking their ARVs anymore because they have given up on life
	Patients find new marriage partners and do not want them to find out that they are on ART
	Pregnancy and wanting to protect the unborn child from ART
	Patients prefer alcohol over taking ART
	Patients have too many lovers and not enough time to take their ARVs

*ARV* antiretroviral, *HSA* Health Surveillance Assistant.

### Participant interpretation

#### Clusters in the healthcare-related region

In relation to *Poor Documentation*, provider participants noted that the date and outcomes (e.g., health status, ART supply given) of patient visits are often not recorded accurately, if at all. This was attributed to a high volume of patients, a shortage of health staff, and a lack of proper filing space. ART providers further realized that while patients may be given their ART, if the visit was not recorded, the patient may be incorrectly labelled as LTFU. Participants noted that some patients provide false contact information to hide from follow-up. Without an accurate address, patients can’t be found and remain LTFU. While improving documentation was rated a feasible issue to address, participants struggled to come up with potential strategies that could be used to address documentation issues.

With respect to the clusters on *Tracing*, transportation challenges emerged. While HSAs mostly walk or use a bicycle, they often face rocky terrains and washed out roads. Some HSAs noted that they do not even live in their catchment areas and so have to travel far to get to areas where they then can start tracing. Interestingly, this was contested by DHO participants who mentioned that HSAs should be living in their catchment areas. A lack of housing options or the need to move with family may help to partially explain this finding. Timing of tracing was also discussed and according to one ART provider: “by waiting more than 2 months to start tracing, a patient is already lost.” Provider participants suggested that tracing should be initiated earlier and patients should be prioritized for tracing. A master list of patients expecting to be seen each week could be generated at each clinic. When a patient doesn’t show up, participants felt that tracing should begin immediately. ART providers suggested that lessons could be drawn from successes with Tuberculosis (TB) tracing.

In relation to *Health Worker Attitudes* patients noted that providers often use exposing language (e.g., ‘ART patients in this line’). Participants generally agreed that enhanced one-on-one counselling could help improve a patient’s overall knowledge of the importance of adherence as well as help to manage fears and expectations.

#### Clusters in the patient-related region

*Social and Financial Support Issues* were also discussed. Many patients referred to strict hospital policies including the need to come to care with a guardian (e.g., family member) as a reason why patients may miss scheduled visits. This would be particularly problematic for patients who have not disclosed their HIV status or who lack a guardian. Interestingly, providers in our study noted that this specific policy was no longer in effect. Being too sick to come to the clinic was the most important and feasible statement to address (data not shown). HSAs spoke to experiences where they traced patients that had been too sick to make it to their appointments. To minimize the likelihood that patients (particularly those without guardians) stop taking ART, the HSAs suggested that they could bring ART directly to the patients in their homes. Patients suggested that there could be a phone number that they can call if they are too sick to come for care. As elaborated by one patient, the hospital can then make alternate arrangements for them (e.g., providing transport). Some patients referred to a lack of food needed to take with ART as a reason why patients may become LTFU. However, other patients suggested that even if food supplement were provided at the clinics, individuals may choose to not come given that others would know where they got their food from and identify them as HIV positive.

Finally, participants discussed the need for education for church leaders as often it is church leaders who discourage patients from taking ART. Having a chaplain at the hospital to provide social and religious support was also suggested as a way to encourage ART adherence and prevent losses to follow-up.

## Discussion

In the present study, we used CM to incorporate both patient and provider perspectives to comprehensively and systematically identify important and feasible issues to address with respect to why patients on ART become LTFU over time. The final 9 cluster concept map consisted of both patient- and healthcare-related clusters. The findings of the present study can inform the development of strategies that work to prevent patients from becoming LTFU including improved tracing efforts.

*Poor Documentation* and *Resources Needed for Effective Tracing* were rated important and feasible clusters to address. Importantly, the quality and accuracy of data (including data on patients who become LTFU) can vary across large longitudinal cohort studies [34,35]. While reliable ART monitoring is critical for measuring patient outcomes and program performance [35,36], data management can be particularly challenging in resource-poor settings [34,35,37-39]. This has been attributed to poor infrastructure, a lack of trained personnel, and clinic characteristics including patient volume [35,38]. Often front line health workers involved in data collection lack a clear understanding as to how the data they collect will be used and how particularly, it is relevant for their day-to-day activities [34,40]. Improving quality data collection, however, may require incentives and ongoing training and supervision for personnel [35,38,40], specifically because data collection can be burdensome [35,37]. Programs with electronic monitoring systems have demonstrated better quality data [35,36] and it is worth noting that DI has improved their electronic system since 2007 although challenges remain. Indeed, ART providers in our study spoke to limited storage space as one reason why files become misplaced and/or lost. Importantly, our findings suggest that while there are patients who truly become LTFU, poor documentation may lead some patients to be incorrectly labelled as LTFU (e.g., the files were lost, visit was not recorded). For example, missing patients may have also transferred to another clinic and therefore may only be LTFU from the perspective of their original clinic [35]. Therefore, identifying where (e.g., which clinics) and when (e.g., data entry, filing) errors occur is a necessary first step in order to determine how it can be addressed although enhanced documentation, improved coordination between clinics (when there are transfers), and effective tracing of missing patients are all necessary components [7,41].

*Tracing* clusters were identified as important and feasible to address in the present study. Participants noted that, based on current definitions and guidelines, tracing may be initiated too late in Malawi. Some studies have suggested that patient retention can be improved when tracing is initiated as soon as possible (dependent on available resources) as this can minimize the likelihood that patients remain LTFU [17,42]. Prioritizing who gets traced and when could mean that fewer patients need to be traced as many patients may ultimately return on their own. Prioritization can be determined through the identification of particular clinical markers that are associated with an increased likelihood of becoming lost or an increased risk of death. In Malawi, for example, risk of death is highest in patients who have recently initiated ART. This suggests that time on ART may be an important way to prioritize patients for tracing [41].

Drawing from the experiences of TB programs, tracers specific to ART could be trained [43] although important differences in the management and perceptions of HIV are important considerations. For example, the largely sexual transmission and labelling of HIV as a ‘moral disease’ makes anonymity and confidentiality of those living with HIV extremely important [22]. Home visits by HSAs may lead to involuntary disclosure of their HIV status [21]. These factors have important implications for how ART tracing efforts should be implemented although as participants in the present study, as well as elsewhere [35,41] have noted, accurate contact information is needed to ensure that patients can actually be found. While the number of patients with cell phones is growing (by 2008, approximately 15% of Malawians were reportedly using cell phones), many patients, particularly those in remote settings, continue to be without working phones [44]. Importantly, while a working phone number is one of the strongest predictors of successfully finding patients [41,45] many still do not return to care [40,42,46,47]. With enhanced training on ART counselling, HSAs may be better equipped to encourage the patients they trace to return to care. Currently, many struggle with questions from patients related to the need for adherence and/or management of side effects.

Participants rated *Social and Financial Support Issues* high on importance. Poor health and feeling sick have been previously associated with stopping ART and/or an increased risk of becoming LTFU [10,48,49]. During interpretation, participants determined that HSAs and guardians can help support patients who are unwell by bringing ART to patients in their homes when they are too sick to travel [50]. The identification of the potentially important role of guardians was, to our knowledge, a finding unique to our study. Disclosure of one’s HIV status has been recognized as a double-edged sword [51] - having the potential to yield needed social support but that may also result in stigmatization and abandonment [51-53]. Through participation in support groups, patients have a safe place to share experiences and support one another which can also strongly motivate patients to adhere to ART [22].

Findings around transport-related costs are consistent with previous studies [10,14,17,18,20]. Patients often have to choose between using their limited income on transport or food for themselves/families [54]. Indeed food insecurity and the perception that ART needs to be taken with food have been shown to influence whether patients can make it to their visits and keep taking their meds [13-15,55,56]. While providing food supplements at the clinics may encourage patients to remain in care [15], this may also deter others, particularly when individuals do not want to be associated with ART. Future research is needed to identify strategies that improve patient retention while supporting patients with logistical challenges that may place them at increased risk for becoming LTFU.

A brief discussion on *Health Worker Attitudes* and *Stigma and Fears* is warranted given rating values. It is worth noting that these clusters included both patient- and healthcare- related statements. Within *Health Worker Attitudes* for example, statements largely speak to patient frustrations with the healthcare they receive particularly around the use of exposing language [21]. Stigma has been previously associated with patients stopping treatment in Malawi [10]. While taking ART can provide patients with a renewed sense of life, allowing them to get back to work and take care of their families, stigma continues to play a powerful role [22]. Experiences of HIV-related stigma can not only affect adherence to ART but may also impact on social aspirations and trajectories linked to a positive identity [21]. While we were surprised that *Stigma and Fears* was not rated higher, participants in the present study may view stigma as an underlying issue rather than a more immediate cause of why patients become LTFU. For example, a patient may seek care further from his/her home for fear of stigmatization, resulting in having a farther distance to travel, thereby increasing transport costs. The latter issue may be seen to be the more immediate issue and as a result, given a higher rating value. In fact, in a recent study, transport costs and other logistical barriers were more commonly reported reasons for why patients become lost when compared to stigma [49]. Healthcare delivery models that acknowledge patient fears related to stigmatization and isolation are critical as these can undermine relationships that are essential for survival [57,58].

There were several limitations in our study. Firstly, while we attempted to include the perspectives of several stakeholder groups, numerous potentially relevant stakeholders with important perspectives may not have been adequately represented including family members of ART patients and religious leaders. Excluding participants without a secondary school education from sorting activities may have biased our final cluster map towards a more highly educated population although it is worth noting that a secondary school education was not required for the other study phases. While sampling was purposive, due in part to feasibility issues, more providers than patients participated in sorting and rating which may further bias our findings more towards a provider perspective. Participant fatigue/sorting burden may have been particularly important during sorting although we attempted to limit the number of statements to be sorted. Rating may have also been impacted by participant fatigue which may help to explain the small range of rating values. Social desirability bias may be present in interpretation activities where participants were asked to interpret results in which many applied to shortfalls in healthcare although participants were informed at the start of the study that all perspectives were valuable. Individuals who chose to volunteer and participate in this study may have been fundamentally different from those who declined to participate, particularly if they have had poor experiences in the past with researchers or their service providers in general. Related to this is the recruitment of patients who were already LTFU. Those that were reachable by phone or in person and agreed to participate may be different from those we could not find. Regardless, differences in participation across individuals we attempted to recruit may impact on the generalizability of our findings to the broader population of interest (e.g., ART patients in Zomba). Finally, while our findings may not be generalizable to individuals outside of Malawi, the concept map presented here identified linkages between various factors that influence losses to follow-up and therefore, may be of interest to ART managers and providers internationally.

## Conclusions

Through the incorporation of patient (including those who were LTFU), provider, and decision-maker perspectives, we determined that individuals can become LTFU for a multitude of patient- and healthcare-related reasons. In general, nine major themes related to why individuals become LTFU emerged and were identified. Poor documentation and issues related to tracing were identified as being particularly important in our context. CM offered a unique approach that combined the strengths of qualitative methodologies of data collection with quantitative methods of analysis to further our understanding of LTFU in ART programs in the Malawian setting. Both patient and provider perspectives were brought to the forefront, and offered insight into potential priority areas for action. Findings of the present study have implications for strategies that maximize patient retention in ART programs including improved documentation of patient visits, enhanced coordination between clinics involved in transfers, and more comprehensive counselling for patients.

## Competing interests

The authors have declared that they have no competing interests

## Authors’ contributions

BR conceived the study, developed and conducted concept mapping activities and generated a first draft. FA conceived the study, assisted with study development, supported CM activities and contributed to the drafting of the manuscript. MvL assisted with the development of the study and supported CM activities in Malawi and assisted with manuscript drafting. AM assisted with the conceptual design of the study. MS provided support during field activities. DCC assisted with conceptualization, study design and development. All authors read and approved the final manuscript.

## Pre-publication history

The pre-publication history for this paper can be accessed here:

http://www.biomedcentral.com/1472-6963/13/210/prepub
